# Continuous movement behavior of humpback whales during the breeding season in the southwest Indian Ocean: on the road again!

**DOI:** 10.1186/s40462-017-0101-5

**Published:** 2017-05-01

**Authors:** Violaine Dulau, Patrick Pinet, Ygor Geyer, Jacques Fayan, Philippe Mongin, Guillaume Cottarel, Alexandre Zerbini, Salvatore Cerchio

**Affiliations:** 1 GLOBICE, Saint Pierre, 97410 Réunion; 2 Parc National de la Reunion, Plaine des Palmistes, 97431 Réunion; 3 Instituto Aqualie, 714/206, Juiz de Fora, MG Brazil; 4 Brigade Nature Océan Indien/ONCFS, Saint-Denis, 97400 Réunion; 5 Marine Mammal Laboratory, Alaska Fisheries Science Center, National Marine Fisheries Service, National Oceanic and Atmospheric Administration, Seattle, WA 98115-6349 USA; 6 Cascadia Research Collective, Olympia, WA 98501 USA; 7 New England Aquarium, Boston, MA 02110 USA; 8 Woods Hole Oceanographic Institution, Woods Hole, MA 02543 USA; 9 Formerly: Wildlife Conservation Society, Bronx, NY 10460 USA

**Keywords:** Humpback whales, Satellite tracking, Reunion, Indian Ocean, Breeding behavior, Movement pattern

## Abstract

**Background:**

Humpback whales are known to undertake long-distance migration between feeding and breeding sites, but their movement behavior within their breeding range is still poorly known. Satellite telemetry was used to investigate movement of humpback whales during the breeding season and provide further understanding of the breeding ecology and sub-population connectivity within the southwest Indian Ocean (SWIO). Implantable Argos satellite tags were deployed on 15 whales (7 males and 6 females) during the peak of the breeding season in Reunion Island. A switching-state-space model was applied to the telemetry data, in order to discriminate between “transiting” and “localized” movements, the latter of which relates to meandering behavior within putative breeding habitats, and a kernel density analysis was used to assess the spatial scale of the main putative breeding sites.

**Results:**

Whales were tracked for up to 71 days from 31/07/2013 to 16/10/2013. The mean transmission duration was 25.7 days and the mean distance travelled was 2125.8 km. The tracks showed consistent movement of whales from Reunion to Madagascar, demonstrating a high level of connectivity between the two sub-regions, and the use of yet unknown breeding sites such as underwater seamounts (La Perouse) and banks (Mascarene Plateau). A localized movement pattern occurred in distinct bouts along the tracks, suggesting that whales were involved in breeding activity for 4.3 consecutive days on average, after which they resume transiting for an average of 6.6 days. Males visited several breeding sites within the SWIO, suggesting for the first time a movement strategy at a basin scale to maximize mating. Unexpectedly, females with calf also showed extensive transiting movement, while they engaged in localized behavior mainly off Reunion and Sainte-Marie (East Madagascar).

**Conclusions:**

The results indicated that whales from Reunion do not represent a discrete population. Discrete breeding sites were identified, thereby highlighting priority areas for conservation. The study is a first attempt to quantify movement of humpback whales within the southwestern Indian Ocean breeding range. We demonstrate a wandering behavior with stopovers at areas that likely represent key breeding habitat, a strategy which may enhance likelihood of individual reproductive success.

**Electronic supplementary material:**

The online version of this article (doi:10.1186/s40462-017-0101-5) contains supplementary material, which is available to authorized users.

## Background

Over the last decade, rapid advancements in tagging technology and tracking tools have improved significantly the ability to remotely investigate animal movement. Comprehensive assessment of movement of wide-ranging species such large marine vertebrates can provide new insights into their temporal and spatial distribution, migratory patterns and habitat utilization, which are essential to promote effective management of highly mobile species [1]. Detailed analysis of animal path may highlight areas particularly relevant to the species life history such as important feeding and breeding sites, where conservation effort should be focused. Investigating movement behavior may also provide important information regarding connectivity between sites and the scale at which protective measures are necessary [2, 3].

Humpback whales (*Megaptera novaeangliae*) typically undertake latitudinal migrations, resulting in a strong and constrained annual cycle, where feeding and breeding activities are spatially and temporally segregated [4]. In the southwest Indian Ocean (SWIO), humpback whales breed along the east coast of Africa, Madagascar and around small oceanic islands, and feed almost exclusively off Antarctica during the austral summer [5]. The SWIO breeding population, originally labelled by the International Whaling Commission (IWC) as breeding stock “C” [6], has been divided into 4 sub-regions [7]: C1, the eastern African coastal waters from South Africa to Kenya; C2, central Mozambique channel islands (Comoros, Aldabra, Eparses islands); C3, coastal waters of Madagascar; and C4, the Mascarene islands, including Reunion, Mauritius and Rodrigues. Thereafter, genetic [8], mark-recapture [9, 10] and satellite tagging data [11, 12] have revealed some level of connectivity between some of the sub-stocks, especially between Comoros (C2) and Madagascar (C3).

The distribution and movement of humpback whales around and between oceanic islands of the southwest Indian Ocean, and their connectivity with other sub-populations of the region, is still poorly known. Sightings have been reported in Mauritius, Rodrigues, Tromelin and Seychelles, without indication of relative abundance, while Reunion Island was shown to have become an important humpback whale breeding habitat in recent years [13]. Humpback whales are present around the island from June to late September, with a peak of breeding activity observed in August [13]. Regional photo-identification comparisons indicate some exchanges between Madagascar and Reunion across years [14], but the level of connectivity within a breeding season is still unknown. Photo-identification studies demonstrate that some whales show relatively high residency around Reunion Island (several weeks), while in the early part of the season, the majority is transient and is thought to reach other destinations during the season [13]. To date, there is no evidence of inter-island movement within the Mascarene’s islands and the eastbound range of the species in the region is not well-defined. The humpback whale turnover observed in Reunion, together with the eastward position of this oceanic island raises the question of breeding habitat range and the role and importance of the Mascarene Islands in the overall SWIO migratory pattern.

At a basin scale, knowledge on habitat use and movement dynamics of humpback whales within their breeding range is limited, particularly in African waters and the Indian Ocean. This is due to limited spatial effort, which is typically restricted to small survey areas, representing a small fraction of the total breeding range [15]. Globally, local photo-identification studies indicate that, for most sites, the majority of whales appear to be transient while only a small proportion of whales remain in the same breeding area over days or weeks [13, 16–18]. The low residency times observed in breeding sites, together with the high proportion of whales seen only once, indicate that the breeding range usually exceeds the spatial scale of survey areas and that whale movements occur over larger geographical scales. A few photo-identification studies have documented long-range movement within the breeding season [16, 19–21] however within-year movement remains relatively difficult to explore using mark-recapture data. In the last decade, satellite tagging has been successfully applied on humpback whales and has revealed detailed movement of whales beyond the range of traditional surveys [11, 12, 22–31]. Even though whale tracks revealed long-range movement of individuals within their breeding range [11, 12, 22, 23, 25–31], with few exceptions [12, 25, 29, 31] much of the current tagging effort has happened at the end of the breeding season to cover the migration back to the feeding areas. Thus, movement strategy in relation to breeding or within the breeding grounds has not been fully investigated.

Specific studies on movement behavior of humpback whales during the breeding season are needed to bring further understanding of their mating system at a large scale. Humpback whales are recognized as polygynous, with males attempting to mate with multiple females to optimize their reproductive success [32, 33]. On the breeding sites, males adopt several alternative tactics to gain access to females: they compete directly with other males through intense physical aggression in “competitive groups” over a central female [34, 35], they escort females with a newborn calf, for post-partum parturition [36, 37] and they display by singing long complex songs [38]. Knowledge of the mating behavior of females is more limited and it is still not clear if they maximize their reproductive success by exercising mate choice, through male songs or by inciting competition among males [39]. Aggregations of singers on breeding sites recall many features typical of a lek mating strategy, where males aggregate in confined areas and engage in competitive displays, for females to visit and choose mates [15, 39, 40]. However, details of the mating system of humpback whales are still debated and the spatial scale at which mating strategy could occur is largely unknown.

In this context, satellite tags were deployed on humpback whales in the middle of the breeding season in Reunion [13]. Based on previous photo-identification studies, we specifically aimed at tagging in early August, during a period overlapping the presence of both transient animals that reach Reunion in the early part of the season, and the apparently more resident animals that predominate in the later season [13]. This was done to achieve two specific objectives. First, we aimed at investigating movement patterns of whales from Reunion, to understand how they relate to the general distribution and movement patterns in the Southwest Indian Ocean. This work was conducted along with other independent satellite tagging studies in the region [11, 12], in an attempt to obtain a broader picture of the population structure and connectivity in the SWIO. Secondly, we aimed at applying a modelling approach to discriminate between transit movements vs. localized meandering movements of whales in order to identify areas where breeding activity (including mating, calving, nursing, etc..) is most likely to occur. This was done to better understand individual movement behavior and the dynamic of habitat use and to gain insight into the breeding ecology of humpback whales over a large spatial scale.

## Methods

### Tag deployment

A total of 15 satellites tags were deployed during the peak of the breeding season off Reunion (55°E33’/21°S07’). Daily surveys were conducted from 31 July to 16 of August 2013 from the northwestern coast of the island on small boats (5 and 8 m in length). Upon sightings, the group composition and behavior was observed and whale flukes and dorsal fins were photographed for individual identification. Whenever possible, the sex and class of each individual within the group was determined on site, from direct observation [12]. A first observation period was maintained until an individual was targeted for tagging, in an attempt to achieve the most representative sample of behavioral category and sexes. Sex and individual behavior class was determined as described in [12]. Individuals accompanied by a young calf were easily distinguished as mother (MC) while receptive (estrous) females could only be identified when being the focal animal in a competitive group. Individuals were considered as male when observed in close association with a mother with calf and referred to as an escort (Esc) or when being the principal or secondary escort (PE and SE respectively) in a competitive group.

Transdermal SPOT5 satellite tags (Wildlife Computers, Redmond, WA, USA) were deployed using a modified pneumatic air gun (Air Rocket Transmitter System, [41]) near the base of the dorsal fin (right or left side). The tags were duty cycled to transmit 18 h daily (9 h on, 3 h off) for 3 months after deployment to acquire high resolution tracking data over the breeding season. On the fourth month, tags duty cycle switched to transmit every other day and on the 5^th^ month every 2 days, in order to prolong battery life. A skin sample of the tagged individual was collected simultaneously to tag deployment, using a Barnett-Panzer crossbow (150 lb) and specific darts equipped with biopsy tips (5x25mm) from Cetadart©.

During the same year, in addition to the tagging operations, dedicated surveys were conducted in Reunion coastal waters over the full breeding season (from June to October 2013) for photo-identification studies. Photographs of the tagged whales (fluke and dorsal fin) were compared to the 2013 photo-identification catalogue to investigate the presence of these individuals in Reunion in the period pre- and post-tagging.

### Molecular sexing

Sex was determined genetically from biopsy samples. DNA was extracted from skin samples using Qiagen DNEasy kits. The ZFX/ZFY region of the sex chromosomes was amplified by polymerase chain reaction [42], separated by electrophorese and visualized under UV-light to discriminates the males (two products) from the female (one product).

### Argos data analysis

Location data were obtained via the Argos System of polar-orbiting satellites (Argos, 1990). Each received location was allocated a Location Class (LC = 3, 2, 1, 0, A, B, Z) referring to decreasing levels of accuracy. Location classes 3, 2, 1, 0 are computed from at least 4 successive uplinks during a satellite pass, with accuracy estimates within 0.250, 0.500, 1.5 km for LC = 3, 2, 1, respectively. No error estimates are available for location class 0 and for those computed from only 3 or 2 satellite uplinks (LC = A and B respectively). Positions of class 3, 2, 1, A are estimated to be accurate to about 2 km, while 0 and B locations are accurate to about 5–10 km [43]. Locations failing the Argos plausibility tests are classified as LC = Z and were not considered in this study.

A speed filter was applied to remove locations corresponding to unrealistic swimming speeds. The threshold value was obtained by computing the swimming speed from the raw data using high quality locations only (LC = 3). The maximum swimming speed obtained was 12.7 km/h, so a threshold of 12 km/h was chosen for removal, which was consistent with speed filter used in other humpback whales tracking studies [11, 12, 22, 23, 27].

#### State-space model

A switching-state-space model (SSSM) was fitted to the filtered data, in order to standardize both the number of locations available per day and the lag time between positions, and to infer animal behavioral state from movement pattern [44, 45]. The SSSM was implemented through the R package bsam using R (v3.1.2. R Core Team) and JAGS (v3.4.0) software. The SSSM model is based on a Bayesian approach, and was fit using Markov Chain Monte Carlo (MCMC) methods. Two chains were run, with a total of 40,000 MCMC samples each. The first 30,000 samples were discarded as a “burn-in” and one every 10th sample was retained to reduce autocorrelation. Posterior distributions for each parameter were based on a final MCMC sample size of 2,000. Different time steps (3, 6, 9 and 12 h) were tested in the SSSM and a 6-h time step (4 positions per day) was chosen to encompass the 3 h off in the duty cycle, while keeping a relatively high resolution in the tracking data. A hierarchical SSSM, which allows for several individuals to be analyzed simultaneous, was used so that model parameters are estimated across multiple tracks [46]. The hierarchical model was run separately for males and females due to potential sex-specific differences in movement patterns. Only individual tracks that provided more than 12 days of data were used in the model. In cases when transmission was interrupted for more than 36 h, the individual track was divided into sub-tracks, so that the model was not constrained to extrapolate positions periods with missing data. Speed and distance travelled was computed from the modelled–derived positions using the “rdist.earth2” function in R.

Whale behavior was inferred from the behavioral mode output of the SSSM. Based on the mean turning angle and autocorrelation in speed and direction, the model provides, for each predicted location, a behavioral mode (b-mode) value ranging from 1 to 2. A behavioral mode <1.25 refers to highly directional and consistent movement and is thus related to “transiting” behavior, whereas a value >1.75, refers to erratic, meandering movement and rather relates to “localized” behavior within breeding habitats [12, 30, 31, 47, 48]. Any locations with a mean behavioral mode between 1.25 and 1.75 were considered as “uncertain” behavior.

#### Environmental data

Depth, slope and sea surface temperature (SST) were extracted for all model-derived locations from raster layers, in QGIS 2.4. SST data were monthly averaged at a spatial resolution of 1° and were obtained from NOAA-POES-AVHRR (available from Bloomwatch 180 data browser). Ocean bathymetry data were obtained from NOAA ETOPO1 and used to produce the slope raster layer, using the slope tool in QGIS. To assess the influence of these factors on the b-mode, linear mixed-effect models (LMM) allowing for both fixed and random effects were run using the NLME package in R. The general model (Model 1) was fit with depth, slope, SST, sex and region (Reunion, Oceanic (>500 m deep waters) and Madagascar) as fixed effects and individuals as a random effect. A spatial autocorrelation function (rational quadratic), available in the NLME package, was included in the model to account for lack of independence of tracking data. Models were then run individually for each of the three regions (models 2, 3 and 4 for Reunion, Oceanic and Madagascar regions respectively), for a separate assessment of covariates within each region and comparison to available data of animal movement around Madagascar [12, 31].

#### Spatial distribution of localized behavior

To identify areas where localized behavior concentrate and as an attempt to identify main breeding sites, a fixed kernel density estimation (KDE) was applied on the positions associated with a localized behavior (bmode > 1.75) and occupancy contours were extracted [49, 50]. The KDE estimates the density distribution of the locations in a grid format from which volume contours, representing the smallest possible area containing a given percentage of the locations, were created [51]. The KDE was performed in R, using the package adehabitatHR, by setting the smoothing factor to 10 km (h = 0.1) and the output grid cell size to 3 km^2^. As the least-square cross validation method provided over-scaled estimates, the bandwidth was set arbitrarily to 10 km as it corresponded to the average distance separating 6 h spaced locations in localized behavior. Volume contours were extracted in 10% intervals, with the 90% contours encompassing most of the localized behavior and the 50% contour considered as corresponding to high-use areas [51]. To avoid over-representation of the tagging site, the kernel analysis was run for each region separately (Reunion, Oceanic and Madagascar).

### Individual movement behavior

To further investigate movement behavior, we identified bouts of localized behavior, hereafter called “localized bouts”. Bouts were defined as consecutive locations associated with a localized behavior and ended when 3 or more consecutive locations classified as “uncertain” behavior, or when 2 or more consecutive locations were classified as in “transit” (adapted from [48]). Time spent and distance travelled within each bout were computed by summing distances between consecutive positions. Time and distance travelled between consecutive bouts of localized behavior were also calculated. A linear mixed effect model was used to assess whether the duration of the localized bouts varied between sex and region (Reunion, Oceanic and Madagascar), while accounting for individual variation.

## Results

### Tagged whales

Fifteen tags were successfully deployed from the 31^st^ of July to the 16^th^ of August 2013. Skin samples were available for 13 of the 15 whales tagged, allowing molecular sexing to be carried out (7 males and 6 females). For the two individuals for which biopsies were missing, gender was inferred from behavioral observation: one whale (tag#120946) was accompanied by a calf and was thus considered a female; the other whale (tag#120950) was identified as a principal escort in a competitive group during the tagging operation and was subsequently observed (photographically recaptured) escorting a mother with a calf, and was thus considered a male. Therefore, the tagged whales included 8 males and 7 females (Table 1). Among the females, all but one were mothers with a calf and the only female without a calf was tracked for only one day. Among males, 2 were observed escorting a mother-calf and 6 were observed in a group of 2 to 4 adult individuals, including 3 competitive groups in which 2 males were identified as principal escort and one as a secondary escort (Table 1).

**Table 1:** Details of the 15 humpback whales tagged off Reunion in 2013, including the number of received locations and number of positions remaining after filtering, tracking time in Reunion, with [*] corresponding to time estimated from both satellite tracking and photographic recaptures, in La Perouse seamount and in Madagascar and overall distance travelled (computed from model-derived position for whales tracked more than 3 days)

Tag#	sex	Behav. Class	Date of first location	Date of last location	Nb of received positions (after filtering)	Tag duration (days)	Last location	Time in Reunion [*] (days)	Time in La Perouse (days)	Time in Madagascar (days)	Distance travelled (km)
120950	M	PE	31/07/2013 09:11	24/08/2013 03:03	234 (194)	23.7	Reunion	23.7 [32]	-	-	625.9
120946	F	MC	02/08/2013 16:56	04/08/2013 19:56	23 (19)	2.1	Reunion	2.1 [12]	-	-	
88719	F	MC	04/08/2013 10:08	06/08/2013 04:08	33 (31)	1.8	Reunion	1.8 [4]	-	-	
112698	F	MC	11/08/2013 17:30	23/08/2013 17:30	150 (98)	12.0	Reunion	12.0 [36]	-	-	281.0
112696	F	MC	15/08/2013 16:56	17/08/2013 01:56	7 (7)	1.4	Reunion	1.4	-	-	
112697	F	MC	16/08/2013 04:54	07/09/2013 15:12	471 (410)	22.4	Reunion	22.4 [52]	-	-	738.3
88721	M	Pair	05/08/2013 06:18	23/08/2013 06:18	241 (237)	18.0	La Perouse Seamount	0.5	17.5	-	790.9
112700	M	Trio	10/08/2013 04:25	05/09/2013 07:25	293 (271)	26.1	St Brandon’s Shoal	3.25 [7]	-	-	2467.5
87776	M	Esc	01/08/2013 16:48	06/09/2013 16:48	363 (299)	36.0	Madagascar	16.0 [18]	12.2	2.7	1956.0
87780	F	MC	06/08/2013 05:07	16/10/2013 08:07	1142 (1127)	71.1	Madagascar	0.5 [5]	-	63.7	5550.0
120948	M	Esc	09/08/2013 14:30	24/09/2013 17:30	628 (611)	46.1	Madagascar	3.5	1.0	36.7	3184.6
88724	M	PE	09/08/2013 04:41	29/08/2013 22:41	324 (312)	20.8	Madagascar	2.75	-	13.5	1774.7
112730	M	SE	09/08/2013 05:46	16/09/2013 20:46	567 (555)	38.5	Madagascar	1.0	-	34.0	3095.1
112719	F	MC	14/08/2013 15:11	13/09/2013 13:03	476 (390)	29.8	Madagascar	11.5	-	14.0	3023.6
112726	M	Pair	15/08/2013 03:34	17/09/2013 02:05	273 (260)	32.9	Madagascar	0.1 [8]	-	21.4	2022.0

Behavior Class: one of two individuals in a pair (Pair), one of two individuals in a trio (Trio), Principal and Secondary Escort in a competitive group (PE and SE), MC (mother with calf of the year), Escort to a mother (Esc). [*]: time estimated from both satellite tracking and photographic recaptures

Pictures of the dorsal fin and fluke were taken from 15 and 8 tagged whales, respectively. Comparisons of photographs taken over the season (June-October 2013) in Reunion indicated that 11 of the 15 tagged whales were photographically recaptured on at least one occasion in addition to the tagging day. Seven whales were first identified from 1 to 18 days before tag deployment and 4 whales were re-sighted from 2 to 23 days after the tag had stopped transmitting.

### Tag duration

The 15 whales were satellite tracked for 1 to 71 days, from the 31 of July 2013 to the 16^th^ of October 2013. Among the 15 tags deployed, 3 stopped transmitting within 3 days or less, while the 12 others lasted for 12 to 71 days (Table 1). Overall, the tags had a mean transmission duration (from first transmission to the last) of 25.7 days (se = 4.8). Although mean tag duration was higher for males (30.4d, se = 3.4 range: 18–46) compared to females (20.2d, se = 9.5, range: 1.5-71), this difference was not statistically significant (Wilcoxon Test W = 42, *p* = 0.1206). Females showed the shortest (1 day) and the longest (71 days) tag duration. When considering only the 12 tags that lasted for more than 3 days, the mean tag duration was 31.6 days (se = 4.5) with similar means between males and females. Among them, 2 tags stopped transmitting for more than 36 h during the tracking period: #120950 was missing 6 consecutive days of data, and #112726 stopped transmitting for 8 days, then 2 positions were received, after which it stopped for another 2 day period and transmitted regularly thereafter.

All tags stopped transmitting within 3 months, so the duty cycle was consistent throughout the tracks. The total number of received locations ranged from 7 to 1142 per tag. Filtering for quality, speed and continent, removed 7.7% of the received locations (Table 1). On average, the locations were acquired at a mean time interval of 1.9 h (SE = 0.06).

### General description of movement

**Fig. 1:**
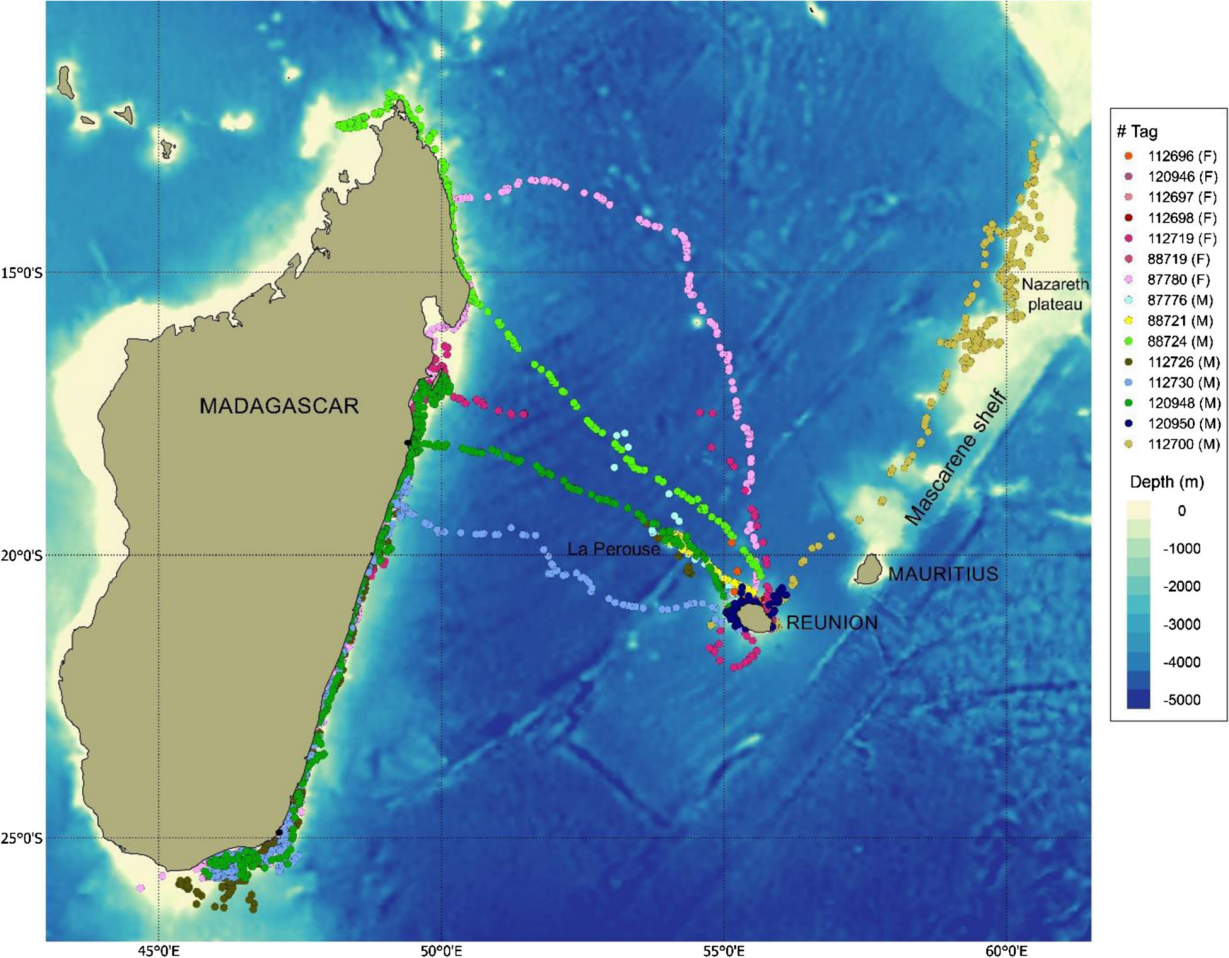
Received Argos locations from whale tagged in Reunion in 2013 (F: Female, M: Male)
- 1 male (tag#88721) stopped transmitting while on *La Perouse* seamount, where it stayed for 17.5 days.
- 1 male moved northeast, travelled along the Mascarene shelf, up to the tip of Nazareth plateau at 12.5°S latitude and turned back south to Saint Brandon shoals, where it stayed for 4 days before the tag stopped (Fig. 1, Table 1).

Individuals tracked more than 3 days travelled a mean distance of 2,150 km (range = 281 to 5,550 km). Three whales, including two mothers with calf and one male, were tracked only in Reunion for 12 to 24 days and 9 whales left Reunion within 16 days of tag deployment (Table 1). Among the 9 tagged whales that left Reunion:

- 7, including 5 males and 2 females (with calf), reached Madagascar with a mean travel duration from Reunion to Madagascar of 7.0 days (range = 3.8 - 17.0 days). Among them, 2 males stopped over for 1 to 13 days in the vicinity of the underwater seamount *La Perouse* (Fig. 1, Table 1), located 160 km northwest off Reunion, and one male passed by the seamount without stopping. Once in Madagascar, whales dispersed along the east coast. One male passed the Northern tip of Madagascar but stopped transmitting shortly (5 days) thereafter.

Therefore, movements from Reunion were generally northwest, with most whales heading to the northeast coast of Madagascar, with the exception of one whale that moved northeast from Reunion. Four of the 7 males that left Reunion after deployment headed to the La Perouse seamount. None of the whales that reached the seamount returned to Reunion. The two females with calves that left Reunion first took a northward heading, and then changed their course toward Madagascar, and thus did not follow the shorter route to Madagascar (Fig. 1).

Although the whales were tagged in Reunion, the majority (51.6%) of the locations were located in Madagascar coastal waters. Mean tracking duration per individual was 8.1 days in Reunion, 10.2 days in La Perouse seamount and 26.6 days in Madagascar (Table 1).

Combining tracking data to photo-identification data collected in Reunion allowed a better estimate of occurrence time around the island. When including dates of first and last photographic captures, the mean occurrence time in Reunion was 15 days (se = 4.7, *N* = 12, range: 0.5-52), with an average of 7 days (se = 1.9, *N* = 9, range: 0.5-18) for whales that moved away from Reunion and 40 days (se = 5.9, *N* = 3, range: 32–52) for whales that stayed in Reunion (Wilcoxon Test: W = 27, *p* = 0.009). The majority (75%) of the tagged whales had left Reunion within 20 days of first sighting, while 25% remained in Reunion for over a month.

### Timing of departure

The timing of departure from Reunion spanned from the 5^th^ to the 26^th^ of August. Male departure occurred over a relatively short period of time: 6 of the 7 males that moved away from Reunion left within a 8 day period between the 10th and the 17^th^ of August. These individual departures occurred within a few hours to 16 days after tagging. Generally, there was a trend to reach lower latitudes up to the end of August, and to move southward starting in early September. From mid-September on, 4 of the 5 whales that were still transmitting had reached the southern coast of Madagascar, while the 5^th^ was in the southeast, travelling southward along the coast.

### SSSM output

**Fig. 2:**
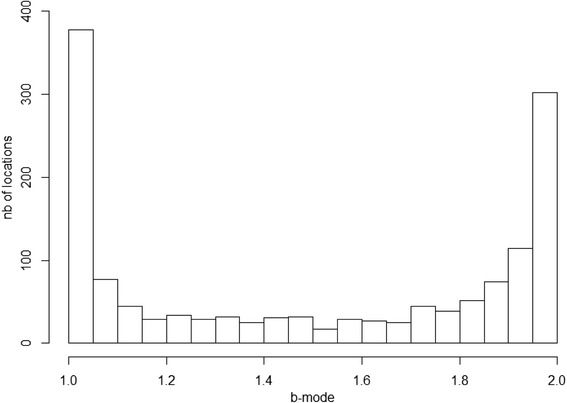
Distribution of locations according to the behavior mode (b-mode) value obtained from the Hierarchical SSSM model run with a 6 h time step

The SSSM model yielded a total of 1435 predicted positions. The b-mode values of the model-derived positions showed a sharp bi-modal distribution (Fig. 2), indicating that the model discriminated well between the two movement behavior categories. Most of the locations (80%) could be assigned a behavior mode, with 39.2% of the locations being assigned a transit behavior (b-mode < 1.25) and 40.7% a localized movement behavior (b-mode > 1.75). Intermediated b-mode values (1.25 - 1.75), classified as “uncertain” behavior, corresponded to 20.1% of the locations.

### Correlation with environmental features

Localized positions occurred mostly in shallow water, at a mean bottom depth of 342 m (se = 29.9) (Table 2). The results of the LMM (model 1), with individuals taken as a fixed effect, indicated that the b-mode values increased significantly with decreasing depth and increasing slope (Table 3). High b-mode values, and thereby localized behavior, were associated with shallow waters and steep slope, which indicates proximity to underwater relief such as insular slope and seamounts. Low b-mode values, referring to transit behavior, tended to occur in deeper waters and flatter sea-bottom, mainly reflecting trans-oceanic movements. Significantly higher b-mode values were obtained in Reunion, where most locations were assigned a localized behavioral state, compared to Madagascar and oceanic habitats, where both localized and transit behaviors occurred. Surface temperature at whale locations ranged from 22.3 to 25.8 °C and did not have any significant effect on the b-mode value (Tables 2 and 3).

**Table 2:** Mean (standard error) speed, depth, slope and sea surface temperature (SST) of the model-derived positions, for all data and by behavior mode (Localized, Transit and uncertain)

	Speed (km/h)	Depth (in m)	Slope (in °)	SST (in°C)
All locations	2.9 (0.06)	720.8 (38.47)	42.3 (0.74)	23.7 (0.74)
Localized behavior	1.8 (0.06)	342.5 (29.90)	55.1 (1.10)	23.6 (0.02)
Transit behavior	4.3 (0.10)	1229.3 (80.77)	30.4 (1.01)	23.7 (0.02)
Uncertain behavior	2.4 (0.09)	412.5 (51.17)	39.5 (1.64)	23.8 (0.04)

**Table 3:** Results of the Linear Mixed Effect model 1, with b-mode as a response variable, individuals as random effect, and Time, Depth, Slope, Sea Surface Temperature (SST), sex and region (Madagascar taken as reference) as fixed variables

Parameter	Estimate	SE	Df	t-value	*p*-value
Intercept	1.6456713	0.4844885	1214	3.396719	0.0007
Time	−0.0004354	0.0008403	1214	−0.518150	0.6044
Depth	0.0000983	0.0000087	1214	11.315819	<0.0001
Slope	0.0001267	0.0000246	1214	5.154891	<0.0001
SST	−0.0081368	0.0191740	1214	−0.424363	0.6714
Sex	0.0804600	0.1133154	10	0.710054	0.4939
Region_Oceanic	0.0454405	0.0446150	1214	1.018504	0.3086
Region_Reunion	0.2930292	0.0454016	1214	6.454153	<0.0001

No significant trend in b-mode was evidenced between males and females overall when running all regions together (model 1, Table 3). However, when running the LMM on each region separately (Additional file 1), sex appeared as a significant variable in Madagascar dataset (model 4), indicating that males showed higher b-mode value, thus more localized behavior, than females. Sex was not a significant variable for the b-mode in either the oceanic or Reunion dataset (models 2 and 3, see Additional file 1).

Whales travelled at a mean speed was 2.9 km/h (Table 2). As a relatively high proportion of transit positions also occurred in shallow habitat, transit movement behavior was further characterized by comparing transit speed and turning angle in shallow vs. deep waters. Whales travelled significantly faster during deep-water transit than shallow-water transit (6.3 km/h, and 3.4 km/h, respectively; Kruskal-Wallis Test *X*2 = 145.9 *p* < 0.001). No difference was found in turning angle between oceanic and shallow waters transit (Kruskal-Wallis Test *X*2 = 0.0092, *p* < 0.923). Thus the transit behavior assigned by the model included two distinct categories of transiting movement: fast deep-water transit and slower transit in shallow habitat.

### Spatial distribution of localized movement behavior

**Fig. 3:**
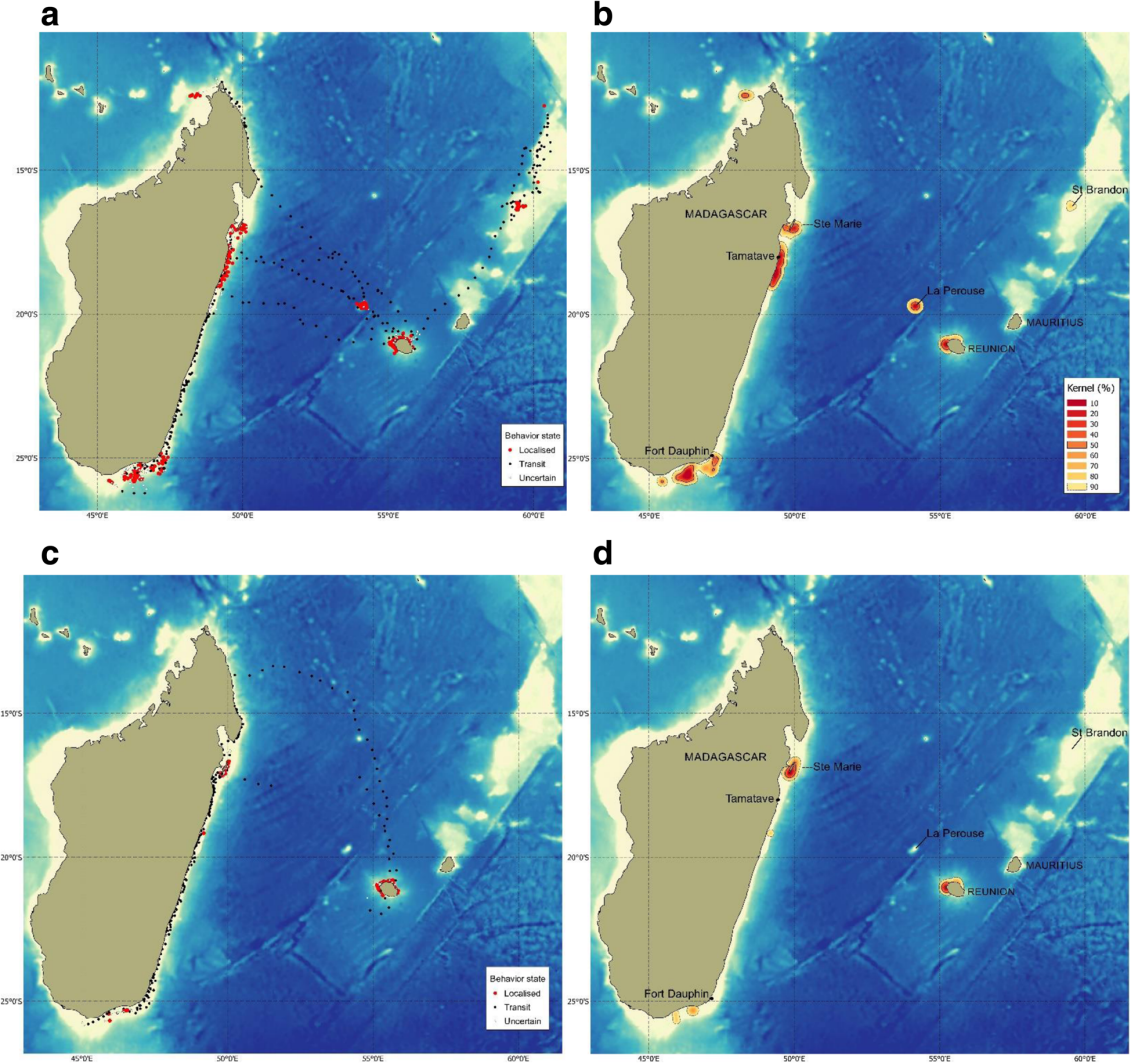
Maps showing the spatial distribution of the model-derived position, with reference to the transit/localized behavior state and the kernel density contours for males (**a** and **b**) and females (**c** and **d**) separately

Locations associated with a localized behavior were highly clustered. Males engaged in localized behavior in 6 main geographical areas: (1) Reunion island, (2) La Perouse Seamount, (3) St Brandon shoal, and off the (4) northern, (5) eastern and (6) southern coast of Madagascar (Fig. 3a). The seamount of La Perouse was identified as a highly used oceanic habitat (kernel density contour <50%), and to a lesser extent, the waters off Saint Brandon shoals, which was used by the single male that traveled northeast from Reunion (Fig. 3b). In Madagascar, the highest concentration of male localized behavior were found off the central east coast (vicinity of the town of Tamatave) and in the south (off Fort Dauphin and on the Madagascar Plateau). In Reunion, the kernel analysis showed that localized behavior was mostly concentrated off the western part of the island (Fig. 3b). Overall, male positions in localized behavior were distributed over a surface area of 35,438 km^2^, 12% of which was around Reunion, 11% over seamount and banks, and 76% off Madagascar (Table 4).

**Table 4:** Surface area (in km^2^) of kernel density contours (90 and 50%) computed from positions in localized behavior, per sex and per region. Number of individuals (n) contributing to each sub-region is indicated

	Males			Females		
	90% contour	50% contour	n	90% contour	50% contour	n
Reunion	4319	1004	4	4285	1073	3
La Perouse	2910	776	2	-	-	
St Brandon	1156		1	-	-	
North Madagascar	2005	384	1	-	-	
East Madagascar	13,047	5116	2	4942	1597	2
South Madagascar	12,001	3252	2	2537		1
Overall	35,438	10,532		11,764	2670	

Female localized behavior was less pronounced and spatially more restricted (Fig. 3c) and covered an overall surface area of 11,764 km^2^ (Table 4). The KDE identified two high-use areas where females engaged in localized behavior (Fig. 3d): Reunion and Ile Sainte-Marie (Madagascar). Therefore, an overlap was observed in the distribution of localized behavior between sexes. No localized behavior of females was observed in oceanic habitat.

### Individual movement behavior

Except for the two females that remained in Reunion and engaged predominantly in localized movement behavior (98-99% of locations), individuals switched alternatively between localized and transit behavior, with 7 to 88% of the positions assigned to localized behavior (Table 5).

**Table 5:** Details on localized behavior (time and %) and on number (n) and mean duration of the localized bouts, mean distance travelled per bouts and number of geographic areas where bouts occurred (Reu: Reunion, SB: St Brandon Shoals, LP: La Perouse Seamount, EastM/SthM/NthM: East/South/North Madgascar), for whales tracked for 12 days or more

Tag#	Sex	Time in localized behavior (hours)	% of positions in localized behavior	n bouts	Mean bout duration (hr) (se)	Mean bout length (km) (se)	n of areas
112697	F	486	98.8	1	486	683	1 (Reu)
112698	F	288	98.0	1	294	281	1 (Reu)
120950	M	270	62.5	3	90 (29.6)	161 (68.9)	1 (Reu)
112700	M	108	17.1	4	27 (21.0)	62 (31.4)	2 (Reu, SB)
112719	F	144	22.4	4	36 (12.7)	115 (31.7)	2 (Reu, EastM)
112726	M	108	19.8	2	57 (27.0)	108 (81.4)	1 (SthM)
112730	M	462	49.7	5	92 (44.4)	234 (102.9)	3 (Reu, EastM, SthM)
120948	M	372	33.5	3	124 (24.6)	256 (77.6)	2 (Reu, EastM)
87776	M	654	75.2	3	218 (108.0)	352 (175.6)	3 (Reu, LP, EastM)
87780	F	126	7.4	4	31 (19.6)	55 (34.5)	2 (EastM, SthM
88721	M	384	87.7	2	192 (180.0)	287 (209.3)	2 (Reu, LP)
88724	M	90	17.9	2	45 (9)	94 (38)	2 (Reu, NthM)

Localized behavior positions were clumped and highly patchy along individual tracks. Individuals engaged in 1 to 5 bouts of localized behavior during the tracking period, with a mean occurrence of 2.8 bouts per track (Table 5). Individual bouts occurred in 1 to 3 of the 6 distinct geographic areas described above, indicating that whales used different sites during the breeding season.

Although they were highly variable, localized bouts comprised a mean number of 17 consecutive localized positions (SE = 3.5) and lasted for 103.1 h (SE = 21.3, range = 6-486) or 4.3 days on average, with a maximum of 20 days (Table 5). The mean bout length was 186 km (SE = 32.3, range = 8-683 km). On average, successive bouts of localized behavior were separated by a mean time interval of 159 h (SE = 33.7, range = 30-750) or 6.6 days on average, during which whales travelled a mean distance of 670 km (SE = 135.1, range = 103-2310).

The LMM analysis showed that the observed variability in the duration of the bouts of localized behavior was mainly due to the random effect of individual variation, and failed to identify any significant difference between sexes and zones.

## Discussion

This study reports on the movement patterns of humpback whales from an oceanic island of the southwest Indian Ocean during the breeding season. Although the sample size was limited, the whale tracks revealed a high level of connectivity between the Mascarene and Madagascar sub-regions. The modelling approach, combined with a cluster analysis, allowed demonstration of the use of yet unknown oceanic habitats and delineation of discrete breeding sites within the whales’ range. Analysis of individual tracks also provided new insights into movement behavior of males and females with calves, suggesting a roaming behavior at a basin scale.

### Tag performance

Individual tracking durations were highly variable, ranging from a few days to a couple of months (max. of 71 days). Tag durations were considerably shorter than the battery endurance, but were consistent with tag longevity reported in previous deployments on humpback whales in the breeding grounds [11, 12, 25, 27, 29, 30]. With a mean tag duration of 31 days (when removing the 3 tags that stopped transmitting within 3 days after deployment), the tags were considered to have performed well knowing that they were deployed in the peak of the breeding season, when whales are usually very active and frequently come into physical contact. Competitive male mating behavior or close interactions between mother and calves increase the risk of tag damage or premature detachment.

### Dispersal and connectivity between sub-regions

Most of the tagged whales dispersed from Reunion and the majority reached the northeast coast of Madagascar. Despite the relatively small sample size, the large proportion of whales that moved to Madagascar indicated a high level of connectivity between Reunion (sub-region C4) and Madagascar (sub-region C3). Photographic capture-recaptures had previously been demonstrated cross-year exchanges between these two sub-regions, with a small number of individuals photographically captured in Madagascar being re-sighted in Reunion 6 to 8 years later [14]; however, direct connectivity between the two regions within the same year had not previously been documented. Therefore, these results represent the first indication of a consistent northwesterly migration stream from Reunion to Madagascar during the breeding season. Inter-island movement was not restricted in gender, as both males and nursing females travelled to Madagascar.

Once in Madagascar, whales dispersed north and south along the coast. This information obtained from whales tagged in Reunion complements other satellite tracking results recently obtained in the southwest Indian Ocean and help improve the understanding of the migration pattern at the basin scale. Satellite tagging of 23 whales in Madagascar, with 12 whales tagged in the northeast in 2012, and 11 whales tagged in the southwest in 2013 (overlapping in tag duration timing with Reunion-tagged whales) indicated no movements eastward to Reunion despite similar sample sizes and tag durations [12]. Satellite tagging also demonstrated direct movement of whales from Comoros archipelago (sub-region C2) to the northwest and northeast coast of Madagascar toward the end of the breeding season [11], while between-years exchanges between these two sub-regions had previously been documented from both photograph and genetic individual recaptures [9]. This suggests that Madagascar (sub-region C3), having a concentration of whales with greater numbers of individuals, may represent a draw for whales visiting both the Comoros (sub-region C2) and Reunion (sub-region C4). Interestingly, some whales tagged in the northeast of Madagascar demonstrated, for the first time, movement from Madagascar to the northeastern coast of Africa (sub-region C1), to Kenya and up to Somalia [12]. Therefore, Madagascar may not necessarily be an end destination for all whales that migrate there, and whales coming from other areas, such as Reunion, might possibly reach other sub-regions after Madagascar.

Although representing only a single individual, the movement of a male northeast from Reunion revealed dispersal across the Mascarene shelf, at least up to the tip of the Nazareth Plateau (12° 36’ S, 60°53’E). The Mascarene shelf is the largest underwater oceanic bank in the Indian Ocean. It extends approximately 2000 km from Reunion to Seychelles and covers an area of over 115,000 km^2^ of shallow water, with depths ranging from 8–150 m. This wide remote bank has never been surveyed for cetaceans during winter and no opportunistic humpback whale data are available for this part of the ocean. Therefore, this result is the first evidence that this large oceanic feature is used by humpback whales during the breeding season, although its importance at a population level is yet to be determined. The Mascarene Islands (Reunion-Mauritius-Rodrigues) might thus represent a stop-over for some whales heading northeast to other yet unidentified breeding habitats. That the male exhibited a meandering course up and down the bank, as opposed to a single directional travel course, as well as localized behavior in the vicinity of St. Brandon, suggests that it may have been searching for other whales or engaging in breeding behavior upon finding conspecifics. In Reunion, the sudden increase in humpback whale frequency suggests a recent use of the island and a possible eastward range expansion of the southwestern Indian Ocean population [13]. Following the ban of commercial whaling, population recovery has been observed in all oceans (see [52] for a review) and in the southwestern Indian Ocean increased abundance has also been reported off Madagascar and Mozambique [17, 53, 54]. Therefore, the observed movement of whales on the Mascarene plateau might also represent exploratory migration to investigate new habitats (or old ones) as a response to population growth.

### Habitat use

Modelling the SSSM output with environmental characteristics showed that behavioral mode was influenced by slope and bottom depth but not by sea surface temperature. Localized movement occurred almost exclusively in shallow waters and tended to be associated with steep bottom, which was consistent with breeding habitat preferences previously described for the species [4, 55–58]. Although mostly observed in coastal waters of Reunion and Madagascar, localized movement was also observed off-shore, over oceanic features such as seamounts (La Perouse) and banks (Mascarene Plateau). The results demonstrated relatively extensive use of La Perouse seamount, located 90 nm northwest of Reunion with an average depth of about 50 m over a surface area of approximately 50 km^2^. The importance of underwater features for migrating humpback whales has recently been demonstrated in the South Pacific [28] and South Atlantic [30]. Satellite tracking revealed a high proportion of humpback whales dispersed from New Caledonia to surrounding seamounts and banks before or during their southward migration [27, 28], and two tagged whales from Gabon travelled along the Walvis Ridge [30]. Other studies have revealed stop-overs on banks and seamounts during the migration back to the feeding grounds, these speculated to be associated with early foraging [11, 12, 23, 30]. In our study, the purpose of localized activity over La Perouse seamount can only be inferred since it was not directly observed. The fact that it occurred in low latitudes (19°S44’), during the peak of the breeding season and over a relatively long period of time (2 whales remained there for 13 and 15 days), would tend to suggest that this underwater feature represents breeding habitat. More data are needed to confirm the use of the seamount, both in terms of density of whales and functionality. A visual (photo-identification and behavioral) and acoustic survey over the seamount of La Perouse is planned to investigate this further.

Transit behavior was not solely associated with deep oceanic waters and also occurred in shallow waters, along Madagascar shelf and over the Mascarene Plateau. Investigating this further, whales travelled significantly faster during deep-water transit (6.22 km/h) compared to shallow water transit (3.9 km/h). This result is consistent with a hypothesis that deep-water transit could be comparable to trans-oceanic migration, while transit in shallow water could rather reflect roving movement, at lower speed, between suitable breeding sites (i.e., searching for localized patches of breeding whales). Mean travel speed during deep-water transit was within the range of speed reported during off-shore migration between feeding and breeding areas [11, 22, 23, 25, 28, 31]. In wintering areas generally, it was previously shown that whales usually travelled at slower speed [11, 22, 25, 31], although changes in movement behavior within and between breeding sites (localized vs. transit behavior) were not accounted for (excepted in [31]).

### Breeding sites

The kernel density distribution showed that the geographic areas utilized by humpback whales when engaged in localized behavior are relatively small, as compared to their total range size. Although the estimated surface areas of these high-use areas are not definitive and require a larger sample size, this study is a first attempt to quantify the spatial scale of highly used sites on the breeding grounds. Localized behavior of females was mostly confined to Reunion and Ile Sainte Marie (East Madagascar), while males used 6 main areas, with Reunion, the Seamount of La Perouse, and areas off the east and south coasts of Madagascar being the most highly used. Given that this is the breeding season and these animals have migrated to this region for breeding, it is reasonable to conclude that these areas represent key breeding habitats. These results were congruent with those of Cerchio et al. [12] that suggested that the same areas off the northeast and south coasts of Madagascar were breeding habitat, particularly for males. Cerchio et al. [12] suggested that these two areas may be differentially used by whales from different sub-regions, because whales tagged in the northeast coast (Sainte Marie) in 2012 used almost exclusively the east coast area, whereas the south coast of Madagascar was used almost exclusively by whales tagged in the southwest (Anakao) in 2013, with little overlap. However, our results from Reunion suggest that there may be more within-season mixing than apparent in that study, at least for some portion of the population.

Although Reunion, Ile Sainte-Marie and Antongil Bay (northeast Madagascar) were known breeding sites [13, 59, 60], the importance of other areas for humpback whales are still poorly understood. While the coastal waters of Madagascar as a whole are identified habitat for the southwest Indian Ocean breeding stock [7], this study and Cerchio et al. [12] revealed new inferences on habitat utilization, with the eastern and southern coast being areas with concentrations of localized movement, and therefore putative breeding habitats. It is noteworthy that although Antongil Bay has previously been established as an active breeding area with concentrations of whales [17, 59, 61], no whales that visited the vicinity of Sainte-Marie or the northeast coast in our study or Cerchio et al. [12] entered the Bay despite its close proximity relative to the movement patterns of the tagged animals. This may be due to shifts in distribution and habitat use, or variations from year to year in habitat utilization, as also suggested by Cerchio et al. [12].

### Movement behavior

Our results indicate that movement patterns from Reunion were highly variable among individuals. However, two trends were observed: a quarter of the whales (*N* = 3) remained around the island for a prolonged time period (32 to 52 days) and the other 75% (*N* = 9) moved to other destinations, after an average of 7 days spent around the island. The apparent residency times of the tagged whales were consistent with the estimates produced from photo-identification data, which indicated that 70% of the whales visiting Reunion were transient, while a small proportion lingers around the island for several weeks [13]. Maximum residence time revealed by the tracking data (52 days) were congruent with those observed each year in Reunion through photo-identification (max of 39–81 in 2009–2013) [13, Globice unpub. data]. Therefore, the oceanic island of Reunion might represent, for some whales, their primary if not sole breeding destination, although the possibility that they reached other areas later in the season cannot be ruled out. Based upon both mark-recapture and tracking data, whales that remained in Reunion during the tracking period showed residency times that were substantially longer than at other breeding areas [17, 18, 62]. However, comparisons with these later studies are difficult as they are based on photo-identification data, and might thus provide less accurate and survey-effort dependent estimates of residency time.

Despite the indication that some whales remain around Reunion, using a focal area during most of the breeding season, the majority of whales showed a larger range. The tracking data revealed that whales showing more transient migratory behavior remained for a few hours to several days around Reunion before heading to other destinations. This roaming behavior was consistent with previous satellite tracking data, revealing wide-ranging movement of humpback whales within their breeding range [11, 12, 22, 23, 25–30]. However, with a few notable exceptions [12, 25, 29, 31], most telemetry studies of humpback whales on breeding grounds were aimed at documenting mainly migration back to feeding grounds, so tags were deliberately deployed toward the end of the breeding season [22–24, 26–30], and thus movement patterns within the breeding areas were only partially revealed.

#### Movement behavior of females with calves

The two distinct types of movement behavior described above were both observed among the four females with calves tracked: two females remained in Reunion for 32 to 52 days whereas the other two migrated to Madagascar. Of the latter, one was photographically recaptured with her calf in Madagascar (Sainte Marie), 16 days after tag deployment in Reunion [Cetamada/Globice, unpub. data.], which confirmed that the female undertook the migration with her calf. The oceanic migration of mothers with new-born calves from Reunion to Madagascar was relatively unexpected. Cerchio et al. [12] also documented the long-range movement of tagged mothers from northeast Madagascar to Kenya, and to Aldabra Island, and mothers with calves were also reported to make long-range oceanic movements in the eastern North Pacific [25] and in the South Pacific [26]. Furthermore, the two maternal females did not take the most direct route to Madagascar but went first north and changed direction, thereby increasing their travel time off-shore. Once in Madagascar they spent most of their time in transit, with one whale travelling up and down the eastern coast, and therefore did not tend to minimize time spent traveling. Similar behavior was noted for mothers tagged along the coasts of Madagascar [12]. Since whales are fasting during the breeding season [3, 63] and given the high energetic demands of lactation, it may be assumed that nursing females would seek to minimize energy expenditure, to promote calf growth and survival [64]. It would thus be expected that nursing females avoid travelling in the middle of the breeding season to save energy. The trans-oceanic movement of females with calves contradicted these assumptions.

Several hypotheses could be put forward to explain such wandering behavior of females with calves. On breeding sites, it has been hypothesized that maternal females minimize interactions with courting males, which maybe energy-consuming, by remaining in shallower waters [61, 65]. If male harassment drives the spatial distribution of nursing females on the breeding sites, it might also affect their movement behavior at a larger scale. Nursing females could thus adopt a wandering behavior and undertake long-range movement to avoid areas with high densities of males. Other alternative explanations can be proposed. Extensive transit behavior could serve to introduce offspring to the location of important breeding sites, supported by the fact that localized behavior of nursing females overlap with those of males. Maternal transmission of migratory routes to the calf during the first year of life has been proposed to explain the strong maternal fidelity to feeding ground and the significant genetic division observed between breeding stocks [8, 66, 67]. As calves generally separate from their mothers during, or shortly before, their second winter [63], information on suitable breeding habitats is more likely to be passed on to new-born calves during their first winter, rather than postponed to when they are yearlings. In the present study, the sex of the calf was not determined, so there is no indication of whether the wandering behavior pattern adopted by some females were influenced by offspring gender. The extensive movements and high level of exercise during early development could also serve to develop musculature and stamina for the calf before the long migration back to Antarctica [68].

#### Movement behavior of males

This study revealed detailed movement of 8 males and demonstrated that although some males seemed to use a single focal area, the majority visited multiple breeding sites during the breeding season (up to 3 during the tracking period). The exploration of more than one breeding areas in a single year may represent a male mating strategy to maximize encounters with receptive females, and is consistent with a polygynous mating system previously described for humpback whales [32, 33, 37]. The patchy distribution of localized behavior tends to suggest that male humpback whales moved and congregated in specific areas of their breeding range. The mean transiting time between successive localized bouts was 6.6 days. Once they switch to localized behavior, males remained in the area for an average of 4 days, with maximum duration of 15 days observed in La Perouse seamount and Reunion. Thus, despite the high variability observed among individuals and geographic areas, the duration of the localized bouts indicated that once males have reached a suitable breeding habitat they generally remained in the area for several days or weeks. The movement pattern of males through several breeding sites would be consistent with the hypothesis of a “floating lek” mating system [15, 39, 40], whereby males rove between sites (as opposed to being territorial as typical in a classical lek system) and congregate in specific area of their breeding range to display and attract females. However, our limited sample size and the absence of movement data from receptive females do not allow us to shed light onto whether male movement behavior is influenced by female distribution or the reverse, with males actively attracting females to specific areas through songs, or a mixture of both. The factors influencing the animal’s decision to remain in an area or move to another also remain unknown, but may be influenced by the competitive environment, the probability of encountering females and the reproductive status of males.

Furthermore, it was observed that males visited each breeding site only once and thus seemed to optimize their movement pattern at a basin scale to avoid back-tracking. No commuting trip was observed between sites and males that reached Madagascar all headed to lower latitudes and then kept a consistent southbound direction (as opposed to nursing females which travelled up and down the coast of Madagascar). These results suggest that spatial movement patterns of these whales within the breeding range may be oriented and governed at a large spatial scale, rather than random, although a larger sample size is needed to make this inference on a population level. This would suggest that whales direct their movement toward known breeding sites and/or that they have the ability to detect suitable breeding habitat from afar. Movement pattern of males in relation to breeding is poorly documented in large marine vertebrates. Roving behavior of males searching for groups of receptive females has been suggested for sperm whales (*Physeter macrocephalus*) and it was predicted that the spatial range of these movements was dictated by the duration of female estrus [69, 70]. Although female humpback whales are not gregarious, their aggregation on breeding sites and the possible asynchrony of estrus may favor this behavior at a basin scale. In other marine vertebrates, post-breeding satellite tracking of green turtle (*Chelonia mydas)* also revealed that males visited multiple breeding grounds and suggested that the observed detour was strategic and aimed fertilizing females at several sites [71].

## Conclusions

This study provides new evidence supporting a high degree of connectivity between the Madagascar and Reunion sub-regions within the same breeding season, further supporting that whales from Reunion, and probably from the Mascarene islands (sub-region C4), do not represent a discrete population. However, some whales showed a high residency time in Reunion, indicating that at least some individuals remain around the island during most, if not the entire, breeding season. The differing movement and residency patterns observed in this study highlight that management and conservation actions need to be defined at both regional and local scales. Similarly, these differences should also be accounted for when estimating population abundances through mark-recapture studies.

Satellite tracking of humpback whale within the peak of the breeding season allowed to identify major breeding sites in Madagascar (sub-region C3) and Mascarene (sub-region C4), thereby highlighting priority areas for conservation. The study also demonstrated the relative high use of oceanic habitats, and documented for the first time the presence of humpback whales, and most probably breeding activity, around La Perouse seamounts and the Mascarene plateau. Cetacean studies have not been conducted in these regions and future studies are recommended to investigate the importance of these newly identified habitats for humpback whales in the southwest Indian Ocean.

The satellite telemetry data revealed that humpback whales dispersed widely within their breeding range, while the state-space models suggested that breeding behaviors were restricted spatially and temporally. Males visited several sites where they displayed localized movements within the SWIO, suggesting a movement strategy at a basin scale to maximize mating. Unexpectedly, females with calves also showed extensive transiting movement, which presumably provides some benefit to the offspring. These new results contribute to a better understanding of the breeding ecology of humpback whales at a basin scale, which could not be previously addressed by traditional photo-identification surveys.

## Additional file

Additional file 1: Results of the Linear Mixed Effect models run for the Madagascar, Reunion and Oceanic dataset separately, with b-mode as a response variable, individuals as random effect, and Time, Depth, Slope, Sea Surface Temperature (SST) and sex (Males taken as reference) as fixed variables. (DOCX 41 kb)

## Abbreviations

KDE: Kernel density estimation
SSSM: Switching state-space model
SWIO: Southwest Indian Ocean
