# Supplementary material for: Continuous movement behavior of humpback whales during the breeding season in the southwest Indian Ocean: on the road again!
Source: Mov Ecol. 2017 May 1;5:11. doi: 10.1186/s40462-017-0101-5 (PMC5410694; doi:10.1186/s40462-017-0101-5)
Supplement: Additional file 1: — Results of the Linear Mixed Effect models run for the Madagascar, Reunion and Oceanic dataset separately, with b-mode as a response variable, individuals as random effect, and Time, Depth, Slope, Sea Surface Temperature (SST) and sex (Males taken as reference) as fixed variables. (DOCX 41 kb) [file 40462_2017_101_MOESM1_ESM.docx]

**Additional file1.**

Results of the Linear Mixed Effect models run for the Madagascar, Reunion and Oceanic dataset separately, with b-mode as a response variable, individuals as random effect, and Time, Depth, Slope, Sea Surface Temperature (SST) and sex (Males taken as reference) as fixed variables.

| **Reunion (model 2)** | | | | | |
| --- | --- | --- | --- | --- | --- |
| Parameter | Estimate | SE | Df | t-value | p-value |
| Intercept | 1.4722635 | 1.7716205 | 259 | 0.831026 | 0.4067 |
| Time | -0.0085251 | 0.0020396 | 259 | -4.179739 | 0.0000 |
| Depth | 0.0001301 | 0.0000152 | 259 | 8.583695 | 0.0000 |
| Slope | 0.0000917 | 0.0000488 | 259 | 1.880664 | 0.0611 |
| SST | 0.0219931 | 0.0760239 | 259 | 0.289292 | 0.7726 |
| Sex_M | -0.0569375 | 0.0969747 | 9 | -0.587137 | 0.5715 |
|  |  |  |  |  |  |
| **Oceanic (model 3)** | | | | | |
| Parameter | Estimate | SE | Df | t-value | p-value |
| Intercept | 4.067010 | 0.7443452 | 391 | 5.463876 | 0.0000 |
| Time | 0.015482 | 0.0021201 | 391 | 7.302378 | 0.0000 |
| Depth | 0.000068 | 0.0000092 | 391 | 7.336949 | 0.0000 |
| Slope | 0.000126 | 0.0000246 | 391 | 5.132541 | 0.0000 |
| SST | -0.127987 | 0.0301874 | 391 | -4.239762 | 0.0000 |
| Sex_M | 0.100885 | 0.2099203 | 7 | 0.480588 | 0.6455 |
|  |  |  |  |  |  |
| **Madagascar (model 4)** | | | | | |
| Parameter | Estimate | SE | Df | t-value | p-value |
| Intercept | 0.4684855 | 0.7300282 | 544 | 0.6417362 | 0.5213 |
| Time | -0.0006951 | 0.0011792 | 544 | -0.5894712 | 0.5558 |
| Depth | 0.0000750 | 0.0000391 | 544 | 1.9176597 | 0.0557 |
| Slope | 0.0000478 | 0.0001133 | 544 | 0.4218774 | 0.6733 |
| SST | 0.0329714 | 0.0292696 | 544 | 1.1264723 | 0.2605 |
| Sex_M | 0.3032996 | 0.0982250 | 4 | 3.0878052 | 0.0367 |
